# Functional Data Analysis Applied to Modeling of Severe Acute Mucositis and Dysphagia Resulting From Head and Neck Radiation Therapy

**DOI:** 10.1016/j.ijrobp.2016.08.013

**Published:** 2016-08-22

**Authors:** Jamie A. Dean, Kee H. Wong, Hiram Gay, Liam C. Welsh, Ann-Britt Jones, Ulrike Schick, Jung Hun Oh, Aditya Apte, Kate L. Newbold, Shreerang A. Bhide, Kevin J. Harrington, Joseph O. Deasy, Christopher M. Nutting, Sarah L. Gulliford

**Affiliations:** * Joint Department of Physics, The Institute of Cancer Research and The Royal Marsden NHS Foundation Trust, London, UK; † Head and Neck Unit, The Royal Marsden NHS Foundation Trust, London, UK; ‡ Department of Radiation Oncology, School of Medicine, Washington University in St Louis, St Louis, Missouri; § Department of Medical Physics, Memorial Sloan Kettering Cancer Center, New York, New York; ∥ Division of Radiotherapy and Imaging, The Institute of Cancer Research, London, UK

## Abstract

**Purpose:**

Current normal tissue complication probability modeling using logistic regression suffers from bias and high uncertainty in the presence of highly correlated radiation therapy (RT) dose data. This hinders robust estimates of dose-response associations and, hence, optimal normal tissue—sparing strategies from being elucidated. Using functional data analysis (FDA) to reduce the dimensionality of the dose data could overcome this limitation.

**Methods and Materials:**

FDA was applied to modeling of severe acute mucositis and dysphagia resulting from head and neck RT. Functional partial least squares regression (FPLS) and functional principal component analysis were used for dimensionality reduction of the dose-volume histogram data. The reduced dose data were input into functional logistic regression models (functional partial least squares—logistic regression [FPLS-LR] and functional principal component—logistic regression [FPC-LR]) along with clinical data. This approach was compared with penalized logistic regression (PLR) in terms of predictive performance and the significance of treatment covariate—response associations, assessed using bootstrapping.

**Results:**

The area under the receiver operating characteristic curve for the PLR, FPC-LR, and FPLS-LR models was 0.65, 0.69, and 0.67, respectively, for mucositis (internal validation) and 0.81, 0.83, and 0.83, respectively, for dysphagia (external validation). The calibration slopes/intercepts for the PLR, FPC-LR, and FPLS-LR models were 1.6/−0.67, 0.45/0.47, and 0.40/0.49, respectively, for mucositis (internal validation) and 2.5/−0.96, 0.79/−0.04, and 0.79/0.00, respectively, for dysphagia (external validation). The bootstrapped odds ratios indicated significant associations between RT dose and severe toxicity in the mucositis and dysphagia FDA models. Cisplatin was significantly associated with severe dysphagia in the FDA models. None of the covariates was significantly associated with severe toxicity in the PLR models. Dose levels greater than approximately 1.0 Gy/fraction were most strongly associated with severe acute mucositis and dysphagia in the FDA models.

**Conclusions:**

FPLS and functional principal component analysis marginally improved predictive performance compared with PLR and provided robust dose-response associations. FDA is recommended for use in normal tissue complication probability modeling.

## Introduction

Normal tissue complication probability (NTCP) modeling uses radiation therapy (RT) dose data, often in combination with clinical and biological data, to construct statistical models of RT-induced toxicity. There are 2 distinct aims of NTCP modeling: (*1*) accurate prediction of toxicity outcomes for individual patients; and (*2*) estimates of associations between treatment covariates and toxicity. Accurate prediction enables clinical decision support (1), treatment plan comparison, treatment modality selection (2), and personalized dose prescription (3). Robust estimates of associations between covariates and toxicity can inform the design of RT planning interventions aimed at reducing toxicity.

A major weakness of many NTCP models is suboptimal dimensionality reduction of the RT dose distribution (reducing the number of variables used to describe the dose distribution from all of the points on the 3-dimensional [3D] dose grid to a small number of summary metrics). To input dose data into statistical models, the 3D dose distribution delivered to an organ at risk (OAR) is reduced to a single or series of scalar metrics, for example, maximum dose or mean dose, or multiple points sampled from the dose-volume histogram (DVH), such as the volume of an OAR receiving at least *x* cGy (V*x*). Ideally, information from each dose level should be explicitly input into the model to prevent loss of potentially important information. However, given the nature of the dose deposition within the patient, adjacent dose levels are very highly correlated (4). This is problematic for many statistical modeling methods, such as the commonly used logistic regression, which often exhibit biased regression coefficients with large standard errors in the presence of collinearity (5). The structure of the correlations is often consistent between patients because the volumes of an OAR receiving adjacent dose levels are highly correlated for all patients. Therefore, if the same or similar treatment techniques are used, this does not necessarily prevent the models from being able to accurately predict outcomes prospectively for new patients. However, it does result in the regression coefficients of the dosimetric covariates being biased and having large standard errors. The apparent regression co-efficients of the dosimetric covariates do not generalizewell to new patients and, hence, should not be used to determine the strength of associations between correlated dose metrics and toxicity, as is commonly done (6).

A small number of studies have attempted to address this issue through using principal component analysis (PCA) to reduce the dimensionality of the DVH data (7-12). However, PCA has been shown to perform poorly when the number of predictors (DVH points) is comparable to, or larger than, the number of observations (patients), as is often the case in NTCP modeling (9, 11). Functional data analysis (FDA) is a statistical framework for analyzing continuous curves rather than discrete measurements (13). Treating an entire curve, for example, a DVH curve, as a single entity removes the problem of correlation (14) and explicitly retains the relationship between points on the DVH curve, which standard, nonfunctional statistical techniques do not capture. Data are represented as curves through the use of basis functions. There are different types of basis functions including a priori fixed bases, such as splines or wavelets, and data-driven bases, for example, functional principal component analysis (FPCA). Functional logistic regression uses functional data to predict binary outcomes. It is well suited to NTCP modeling because of the continuous nature of DVH curves and the binary nature of toxicity endpoints. Functional logistic regression has recently been applied to NTCP modeling by Benadjaoud et al (15), using FPCA (16) for dimensionality reduction of the DVH data. However, FPCA (and nonfunctional PCA) is unsupervised (it does not use outcome data), which may be a limitation for NTCP modeling. The FPCA components with the most variance in the RT dose data may not be the ones that are most strongly associated with the toxicity outcome of interest. Functional partial least squares regression (FPLS) (17, 18) is a supervised analogue of FPCA. It overcomes this limitation through generating uncorrelated covariates (FPLS components) in the linear space of the predictors, accounting for the correlation between those predictors and outcome, in this case toxicity. As partial least squares regression (and FPLS) uses the outcome (toxicity) data in establishing the components, it often outperforms PCA (and FPCA) in prediction tasks (19). However, because of the inclusion of outcome data, it is more susceptible to overfitting.

In this study we applied FPLS and FPCA to NTCP modeling of severe acute mucositis and dysphagia. We compared our novel application of FDA with nonfunctional penalized logistic regression (PLR) models. The aims of this study were to (*1*) determine whether using FPLS or FPCA to reduce the DVH data would improve predictive performance compared with PLR; and (*2*) assess whether FPLS or FPCA would lead to more robust estimates of associations between DVH data and toxicity than PLR.

## Methods and Materials

### Patient data

Data from 351 head and neck RT patients, enrolled in 1 of 6 different clinical trials (20-24) (International Standard Randomised Controlled Trial Number 81772291), were used to train and internally validate severe acute mucositis and dysphagia NTCP models. Data from the same patients were used for the modeling of both toxicities. This dataset is described in Appendix A (available online at www.redjournal.org) and the publication (25). Mucositis and dysphagia were both consistently scored for all studies using the Common Terminology Criteria for Adverse Events version 2.0 instrument (26) or version 3.0 instrument (27). The mucositis and dysphagia grading systems are nearly equivalent in both versions. Both toxicities were recorded, prospectively, for all patients prior to the start of RT, weekly during RT, and at 1 to 4 and 8 weeks after RT by head and neck cancer specialists trained in the use of the scoring systems, using standard trial protocols. The toxicity outcome was defined as the peak grade of toxicity, dichotomized into grade 3 or worse (severe) and less than grade 3 (non-severe). Grade 3 mucositis corresponds to confluent mucositis and grade 3 dysphagia corresponds to feeding-tube dependence for >24 hours. Patients with baseline toxicity were excluded from the analysis. To attempt to reduce bias at the expense of statistical power, patients with any missing toxicity scores and a peak score below 3 were excluded from the analysis. A detailed justification for this approach is provided in Appendix B (available online at www.redjournal.org). Of the 351 patients, 183 met the inclusion criteria for mucositis modeling (severe mucositis incidence, 73%) and 179 met the inclusion criteria for dysphagia modeling (severe dysphagia incidence, 66%). Ninety head and neck RT patients treated at Washington University School of Medicine in St Louis with acute dysphagia data available were used as an external validation cohort for the dysphagia models (severe dysphagia incidence, 48%). In this cohort severe acute dysphagia was defined as the requirement for percutaneous endoscopic gastrostomy (PEG) tube insertion. It should be noted that there was a slight difference in the scoring systems because of the data available. All centers involved in treating patients included in this study used a reactive approach to PEG insertion, that is, delaying insertion until deemed clinically necessary.

Induction chemotherapy (yes or no), concurrent chemotherapy regimen (cisplatin, carboplatin, 1 cycle of cisplatin followed by 1 cycle of carboplatin, or none), definitive versus postoperative RT, primary disease site (grouped into oropharynx or oral cavity, nasopharynx or nasal cavity, hypopharynx or larynx, parotid gland, and unknown primary), age, and sex were also included as covariates in the models. Concurrent chemotherapy was administered in 2 cycles, on day 1 and day 29 of RT. A comparison of the clinical covariate data in the training and external validation datasets is provided in Appendix C (available online at www.redjournal.org).

### RT dose data

The extended oral cavity (25) and pharyngeal mucosa (described in Appendix D) were contoured by clinical oncologists and used as OARs in the mucositis and dysphagia models, respectively. The physical dose distribution was converted to the fractional dose distribution (physical dose delivered in each fraction). This has been shown to be appropriate for NTCP modeling of acute toxicity (28) as the toxicities often develop before the total dose is administered. The fractional dose distribution was described by the normalized cumulative DVH. Preliminary work indicated that corrections for different fractionation regimens based on radiobiological models made negligible difference to the results. This is because the fractionation regimens used (Appendix A; available online at www.redjournal.org) were similar. An alternative approach would be to use the cumulative dose delivered up to the appearance of the toxicity endpoint. However, treating clinicians’ subjective choice of when to initiate a feeding-tube intervention would lead to substantial noise in the cumulative dose delivered up to the time of intervention.

### PLR model

For the nonfunctional model, the fractional DVH curves were discretely sampled from 0.2 Gy to 2.6 Gy at 0.2 Gy intervals. This sampling was chosen to encompass the entire range of OAR doses with enough granularity to capture the shapes of the DVHs. These DVH measurements were input into a PLR model along with the clinical covariates. Penalization was performed using the least absolute shrinkage and selection operator (LASSO) (29). LASSO reduces the magnitude of the regression co-efficients, setting some to 0, to prevent overfitting. In the context of correlated variables, it reduces the impact of multicollinearity. The penalization strength was selected by 10-fold cross validation with the value producing the highest average (over all of the cross validation folds) area under the receiver operating characteristic curve (AUC) on cross validation selected.

### Functional data analysis

The fractional DVH curves (sampled from 0 Gy to 2.60 Gy in 0.01-Gy intervals) were represented using penalized FPCA (16, 30) and penalized FPLS (17, 31) basis functions. FPCA is a dimensionality reduction technique that represents the functional DVH data as orthonormal vector components explaining the maximum variance between patients in the DVH curves. The orthonormality constraint removes the collinearity in the dose metrics used for subsequent modeling and, hence, overcomes the limitations associated with modeling collinear data. The functional principal components represent the functional DVH data (normalized volume as a function of dose, *d* for patient *i*), *V_i_*(*d*), as the sum of the eigenfunctions, *ξ_k_*(*d*), weighted by their coefficients, *c_ik_*:

(1) $$V_{i}(d)-\mu (d)=\sum_{k=1}^{\infty }c_{\mathrm{ik}}\xi_{k}(d)$$

where μ(*d*) is the mean *V*(*d*) and *c_ik_* describes the score for functional principal component *k* for the DVH of patient *i* and is given by:

(2) $$c_{\mathrm{ik}}=\int \left(V_{i}(d)-\mu (d)\right)\xi_{k}(d)\mathrm{dd}$$

The eigenfunctions, ${\left\{\xi_{k}(d)\right\}}_{k=1}^{\infty }$, and their corresponding eigenvalues (describing the amount of variance explained by each eigenfunction), *λ*_1_ ≥ *λ*_2_ ≥ …, are determined by eigendecomposition (factorization into eigenvalues and eigenvectors) of the covariance operator, Σ, where:

(3) $$\sum \left(d_{1},d_{2})=\mathrm{Cov}[V(d_{1}),V(d_{2})]=E[(V(d_{1}\right)-\mu (d_{1})){\left(V(d_{2})-\mu (d_{2})\right)}^{T}]$$

in which *d*_1_ and *d*_2_ are two different dose levels and E is the expected value. *V*(*d*) can be approximated by a small number of principal components, *k_n_*, assuming that *c_ik_* = 0 for *k* > *k_n_*, to achieve dimensionality reduction to a small number of basis functions efficiently describing the variation between patients in the DVH data:

(4) $$V_{i}(d)\approx \mu (d)+\sum_{k=1}^{k_{n}}c_{\mathrm{ik}}\xi_{k}(d)$$

The eigenfunctions and their coefficients can then be used in subsequent analyses. The FPCA components can be used to estimate a toxicity outcome for patient *i, y_i_*, using a functional linear model (30, 32):

(5) $$y_{i}=\alpha +\int \beta (d)V_{i}(d)\mathrm{dd}+\varepsilon_{i}$$

where α is the intercept and *ε_i_* is a centered random error.

When FPCA is used to describe the DVH data, *β*(*d*) represents a “weighting function” describing the amount of variation between patients at all dose levels on the DVH. It can be approximated by *k_n_* eigenfunctions:

(6) $$\beta (d)=\sum_{k=1}^{\infty }\beta_{k}\xi_{k}(d)\approx \sum_{k=1}^{k_{n}}\beta_{k}\xi_{k}(d)$$

An estimate of the response, *ŷ_i_*, can be made using the following [with the derivation described in a previous publication (30)]:

(7) $${\hat{y}}_{i}=\alpha +\int \beta (d)V_{i}(d)\mathrm{dd}\approx \alpha +\sum_{k=1}^{k_{n}}{\hat{\beta }}_{k}c_{\mathrm{ik}}$$

where

(8) $${\hat{\beta }}_{\left(1:k_{n}\right)}=(\frac{c_{.1}^{T}y}{{\mathrm{n\lambda}}_{1}},\ldots ,\frac{c_{.k_{n}}^{T}y}{{\mathrm{n\lambda}}_{k_{n}}})$$

The model was fit to the data, placing penalization on the curvature (second derivative) of the eigenfunctions, by:

(9) $$\hat{y_{i}}=\xi_{k}{\left(\xi_{k}^{T}\xi_{k}+r\xi_{k}^{T}P\xi_{k}\right)}^{-1}\xi_{k}^{T}y_{i}$$

where *r* is the amount of penalization, **P** is the vector (0, 0, 1) that defines the penalty matrix such that the second derivative (curvature) is penalized, and *y_i_* is the actual outcome (toxicity) data for patient *i*. The choice of which components to include (within the first 5 components) and the magnitude of the roughness penalty, *r*, to apply (selected from a set of values in the range from 0 to 1350) to best estimate the toxicity outcomes were determined using model selection criteria (MSC) (16) with the Bayesian information criterion:

(10) $$\mathrm{MSC}(k_{n})=\log\;[\frac{1}{n}{\sum_{i=1}^{n}\left(y_{i}-\hat{y_{i}}\right)}^{2}]+\frac{\log(n)k_{n}}{n^{2}}$$

where *n* is the number of patients. This penalizes the model complexity to reduce overfitting. Models with different values of *r* and *k_n_* were generated, and the combination of values that minimized MSC was selected. The FPCA or FPLS components included affect the smoothness of the estimate of the *β*(*d*) function as the dominant mode of variation tends to be smooth and roughness tends to increase for subsequent modes of variation, in part because of the orthogonality constraint.

FPLS is similar to FPCA but uses the response (toxicity) data in constructing the FPLS components (17), ${\left\{{\tilde{\xi }}_{k}\right\}}_{k=1}^{\infty }$, to establish orthogonal components that have maximum covariance to the response. This is achieved through maximizing the squared covariance between *V_i_*(*d*) and the response, *y_i_*, with the constraint that all components are mutually orthogonal (31). This takes the place of the eigendecomposition used for FPCA, described in equation 3. The iterative algorithm used to compute the FPLS components was described previously (33). When FPLS is used for dimensionality reduction of the DVH data, *β*(*d*) can be interpreted as a data-driven weighting function for the importance of each dose level in causing severe toxicity. It is important to consider that, as this is a data-driven approach, the weighting function is an estimate of the “true weighting function” over the range of available data and is influenced by the structure (ie, distribution in dose-volume space) of the available data. MSC was performed for the FPLS analysis in the same manner as for FPCA. The FPCA and FPLS analyses were bootstrapped with 2000 replicates to assess the uncertainty in the shapes of the components.

The optimal FPCA and FPLS components (those producing the lowest MSC) were used as basis functions as input into functional logistic regression (34, 35) models (functional principal component—logistic regression [FPC-LR] and functional partial least squares—logistic regression [FPLS-LR]) along with the (nonfunctional) clinical covariates. The functional logistic regression model describes the probability of patient *i* having severe toxicity, *P*(*y_i_* = 1), and is given by:

(11) $$\begin{array}{l} \ln\frac{P(y_{i}=1)}{P(y_{i}=0)}=\alpha +\sum_{j=1}^{p}\beta_{j}Z^{j}+\int \beta (d)V_{i}(d)\mathrm{dd}\approx \alpha \\ \;+\sum_{j=1}^{p}\beta_{j}Z^{j}+\sum_{k=1}^{k_{n}}\beta_{k}c_{\mathrm{ik}} \end{array}$$

using the substitution for the functional linear model described in equation 7, where α is the intercept and ${\left\{Z^{j}\right\}}_{j=1}^{p}$ are the nonfunctional covariates with regression coefficients ${\left\{\beta_{j}\right\}}_{j=1}^{p}$. Maximum likelihood estimation of the regression coefficients was performed using iteratively reweighted least squares.

### Model comparisons

The predictive performance and generalizability of the models (addressing aim 1) were assessed in terms of discrimination, calibration, and overall performance on internal validation, as well as additionally for the dysphagia models on external validation. The discriminative abilities of the models were assessed using the AUC. Calibration was evaluated by the slope and intercept of a logistic regression model of the actual toxicity against the predicted probability of severe toxicity (36, 37). Overall model performance was measured using the Brier score (BS) (38). It is defined as:

(12) $$\mathrm{BS}=\frac{1}{N}\sum_{t=1}^{N}{\left(p_{t}-y_{t}\right)}^{2}$$

where *p_t_* is the predicted probability, *y_t_* is the actual outcome, and *N* is the number of predictions. The score takes a value between 0 and 1, with lower values indicating better performance.

For the internal validation, the performance metrics were “corrected for optimism” using bootstrapping with 2000 replicates (39). The optimism-corrected performance metrics, *M_corrected_*, were calculated by:

(13) $$M_{\mathrm{corrected}}=M_{\mathrm{apparent}}-O$$

where *M_apparent_* is the performance metric, for example, AUC, calculated using all of the training data to both fit the model and evaluate its performance, and the optimism, *O*, is given by:

(14) $$O=\frac{1}{B}\sum_{b=1}^{B}\left(M_{b,\mathrm{boot}}-M_{b,\mathrm{orig}}\right)$$

where *B* is the number of bootstrap replicates, *M_b,boot_* is the performance metric calculated using the bootstrap dataset *b* to both fit and evaluate model performance, and *M_b,orig_* is the performance of the model fit using the bootstrap dataset *b* evaluated on the original dataset. This provides an unbiased estimate of internal validity, penalizing for over-fitting. Model hyper-parameter tuning, such as the selection of the amount of penalization for the PLR models and the selection of components and penalization for the FDA models, was performed for each bootstrap replicate. This prevents any “data leakage” from the training data into the internal validation data. The dysphagia models were used to predict severe dysphagia probability for the external validation cohort. Those predictions were compared to the actual PEG-dependence data for the cohort and the same performance metrics calculated. The uncertainties of the odds ratios (addressing aim 2) were assessed by calculating bootstrapped 95% confidence intervals with 2000 replicates. Statistical analysis was performed using the statistical computing R language version 3.2.4 (40) and the fda.usc version 1.2.2 (41), glmnet version 2.0 (42), rms version 4.5 (43) and val.prob.ci.2 (44) packages.

## Results

For FPCA, the variances in the DVH data explained by the first 5 FPCA components were 80.8%, 12.5%, 3.7%, 1.2%, and 0.6% for mucositis and 70.8%, 14.5%, 5.6%, 4.4%, and 1.6% for dysphagia. For FPLS, the variances explained by the first 5 FPLS components were 78.1%, 16.9%, 2.0%, 2.5%, and 0.6% for mucositis and 76.2%, 8.6%, 11.2%, 2.7%, and 1.3% for dysphagia. The model selection resulted in the first 2 components being selected for the FPCA and FPLS mucositis models and only the first component being selected in both of the dysphagia FDA models. Penalization of 1342 was chosen by the model selection for the mucositis FPCA model, 0 for the mucositis FPLS model, and 1350 for both of the dysphagia FDA models.

**Fig. 1:**
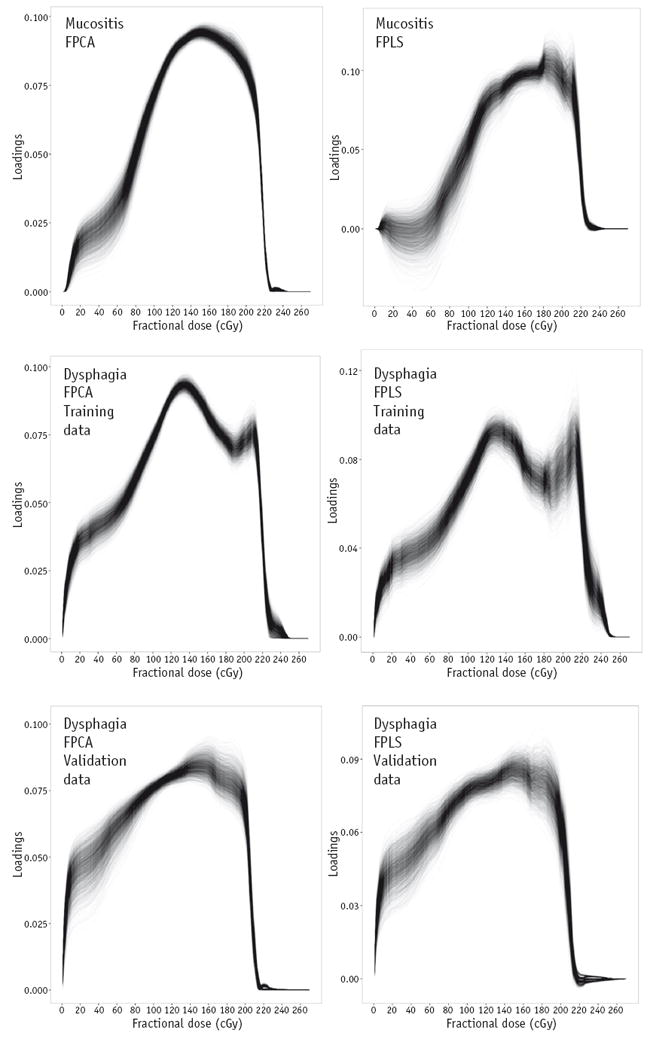
First functional principal component (left column) and first functional partial least squares component (right column) for mucositis training (top row), dysphagia training (middle row), and dysphagia external validation (bottom row) data bootstrapped with 2000 replicates. Each line represents 1 bootstrap sample. The functional principal components show the variance in the patient dose-volume histograms over the range of dose levels. The functional partial least squares components show the covariance between the patient dose-volume histograms and toxicity outcomes over the range of dose levels. Note that the components for the validation data set are shown for comparison with the training data and were not used in any of the model training or validation tasks. *Abbreviations*: FPCA = functional principal component analysis; FPLS = functional partial least squares regression.

Figure 1 shows the first FPCA and FPLS components for the mucositis and dysphagia models. Bootstrapping the FPCA and FPLS indicated that the shapes of the first FPCA and FPLS components were very similar irrespective of the random sample of patients selected. There was a general trend that the FPCA and FPLS loadings increased with increasing dose and sharply decreased to 0 at the maximum dose. The FPCA components indicate that the variation between patients in the volume of OAR irradiated to a certain dose level increased with increasing dose level. The same trend in the FPLS components indicates that higher doses were more strongly associated with severe toxicity. The decrease in the first FPCA and FPLS component loadings at around 1.8 Gy (Fig. 1) for the dysphagia training data is indicative of reduced variation in this region of the DVHs between patients. This is likely to be because most of the variation in the pharyngeal mucosa dose distribution between patients is related to the variation in volume of overlap of the 2 different planning target volumes (whose prescription dose levels correspond to the positions of the 2 peaks in the FPCA and FPLS components) with the pharyngeal mucosa.

For the PLR, FPC-LR, and FPLS-LR modeling, oropharynx or oral cavity and no concurrent chemotherapy were removed as covariates to prevent perfect collinearity (correlation matrices are shown in Appendix E; available online at www.redjournal.org). Odds ratios for other primary disease sites are thus relative to oropharynx or oral cavity, and odds ratios for concurrent chemotherapy are relative to no concurrent chemotherapy.

Regarding aim 1, the predictive performance of the 3 different mucositis and dysphagia models, as assessed by internal and external (for dysphagia) validation, is displayed in Table 1. The mucositis models had modest (PLR and FPLS-LR) or modest to good (FPC-LR) discriminative ability [using the interpretation previously described (45)] on internal validation. The discriminative abilities and overall performances of the FPC-LR and FPLS-LR models were marginally better than the PLR model. Calibration was relatively poor for all of the models, with the FPC-LR and FPLS-LR models overfitting the data (calibration slope <1) and the PLR model underfitting the data (calibration slope >1). The underfitting exhibited by the PLR models was likely due to over-shrinkage of the regression coefficients by the LASSO penalization caused by high multicollinearity. It should be noted that the “correction for optimism” may have improved the calibration of the PLR models, as they underfit the data.

**Fig. 2:**
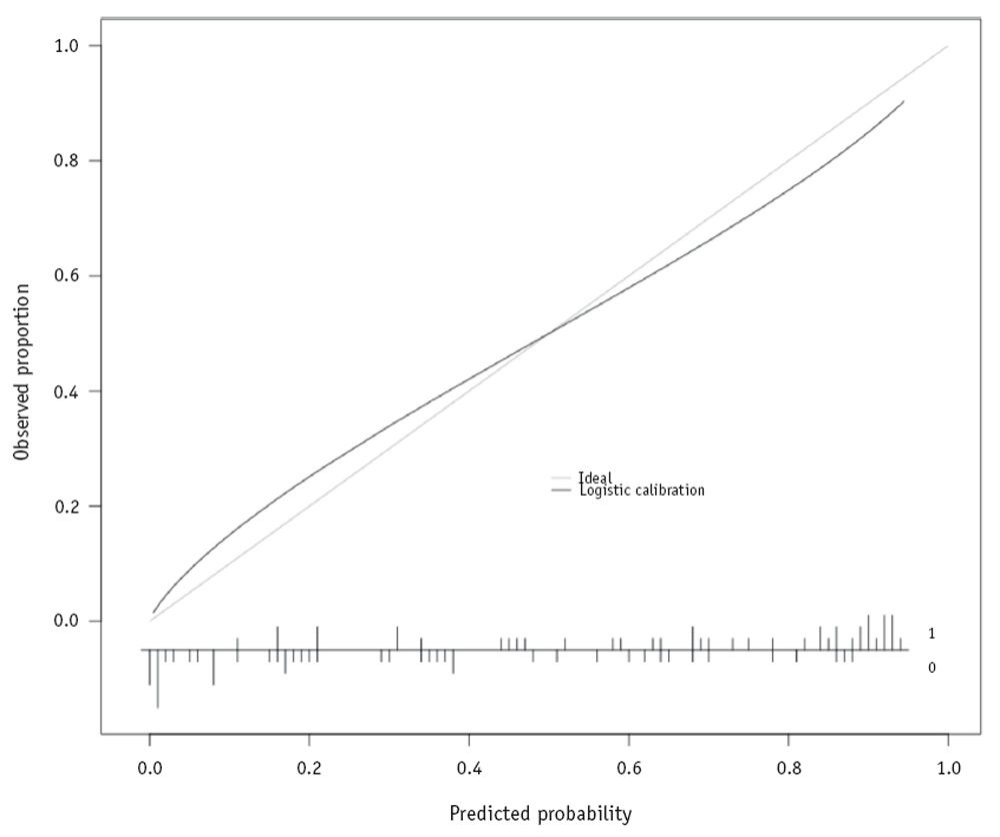
Logistic calibration curve of the functional partial least squares—logistic regression dysphagia model predictions against actual toxicity outcome for the external validation data. The relative frequency distribution of the raw predicted probabilities, along with the actual outcome (where 0 indicates non-severe dysphagia and 1 indicates severe dysphagia), is displayed at the bottom of the figure.

The discrimination and calibration of the dysphagia models were better than the mucositis models. All 3 dysphagia models had good discriminative ability on internal validation. The discriminative abilities of all 3 models increased on external validation, with the PLR model showing good to excellent discrimination and the FPC-LR and FPLS-LR models showing excellent discrimination. The overall performance of all of the models was similar, both on internal validation and on external validation. Calibration of all of the models on internal validation was modest, with the PLR model under-fitting the data and the FDA models overfitting the data. The FPC-LR and FPLS-LR models had substantially better calibration than the PLR model on external validation. The FPLS-LR model had marginally better calibration than the FPC-LR model on external validation. A logistic calibration curve for the external validation of this model is shown in Figure 2. The curve lies close to the identity line, indicating good model calibration on external validation.

Concerning aim 2, the results of the bootstrapped penalized and functional logistic regression odds ratios are shown in Tables 2-4. The odds ratios for the covariates in the PLR models were often set to 1 by the LASSO penalization. In the mucositis and dysphagia PLR models, none of the covariates was significantly associated with severe toxicity. Conversely, there was a significant association between the first FPLS component and severe toxicity in the mucositis and dysphagia FPLS-LR models. The first FPCA components were not significantly associated with severe mucositis or dysphagia. Compared with the first FPLS components, slightly less weight was given to the higher doses (Fig. 1). It should be noted that the sign of the FPCA component loadings is arbitrary, so the fact that the odds ratios are <1 does not indicate that there is an inverse correlation between RT dose and severe toxicity.

None of the clinical covariates was significantly associated with toxicity in the mucositis models. Concurrent cisplatin was significantly associated with severe acute dysphagia in the FPC-LR and FPLS-LR models but not in the PLR model. None of the clinical covariates was significantly associated with severe toxicity in either of the PLR models.

## Discussion

Our results show that FPC-LR and FPLS-LR produced models with marginally better discrimination and overall performance than PLR and superior calibration (aim 1). They also show that FPCA and FPLS are appropriate methods to provide robust estimates of dose-response associations, to inform RT planning, in the presence of highly correlated DVH data (aim 2). We, therefore, encourage the use of FDA methods in future NTCP modeling studies. We suggest that our externally validated dysphagia FPLS-LR model is suitable for clinical decision support. To our knowledge, it represents the severe acute dysphagia model with the best predictive performance to date. Previous models of severe dysphagia during or shortly after RT that measured discrimination had AUC values of 0.62 (46) and 0.74 (47). These studies did not perform external validation. The mucositis FPC-LR model had the best performance on internal validation and should be externally validated to determine its potential to aid clinical decision making. Both models are available at https://github.com/jamiedean/fda-ntcp-models.

The shapes of the first FPLS components indicate that both severe mucositis and dysphagia are most strongly associated with the volume of the oral cavity or pharyngeal mucosa receiving high and intermediate fractional doses (greater than approximately 1.0 Gy). Therefore, RT planning interventions aiming to minimize the incidence of severe acute mucositis and dysphagia should minimize the volumes of oral cavity and pharyngeal mucosa receiving high and intermediate fractional doses, without compromising other aspects of the plan, such as target coverage. Although this is intuitively unsurprising, many RT planning protocols, such as Radiation Therapy Oncology Group (RTOG) 0912, RTOG 0920, and RTOG 1216, set planning objectives based on OAR mean doses, which give equal importance to low doses and high doses. This suboptimal approach is likely taken because of the common use of mean dose to achieve dimensionality reduction in studies aiming to elucidate dose-response relationships. The first FPCA components, which are unsupervised, had similar shapes to the first FPLS components, which are supervised, suggesting that, for this dataset, the variation in severity of toxicities is related to the variations in the DVHs. This suggestion is further supported by the fact that the MSC for FPCA selected the first FPCA component (the one describing the most variation in the DVH data). This will not necessarily be the case for all datasets. The variations in the bootstrapped first FPLS components are slightly wider than those of the first FPCA components (Fig. 1) because of the presence of patients who did not follow the general dose-response trend (ie, received lower doses but had severe toxicity and vice versa). The substantial penalization of the PLR odds ratios (many often being set to 1) shows the limitations of using PLR models to infer associations between correlated dosimetric covariates and toxicity, and hence, we do not recommend its use in this context. Unlike the FDA models, the PLR models were unable to identify that high doses, greater than approximately 1.0 Gy/fraction, had higher correlations with toxicity than low doses, as would be intuitively expected.

The FDA models were also able to identify an association between concurrent cisplatin and severe acute dysphagia. The associations between cisplatin and dysphagia in the PLR model were not significant. This may be due to the fact that concurrent chemotherapy was correlated with the DVH metrics because of patients with parotid gland primary tumors (who receive unilateral, rather than bilateral, irradiation and, hence, lower pharyngeal mucosa doses) not receiving concurrent chemotherapy. The number of patients receiving concurrent carboplatin or a combination of cisplatin and carboplatin was low (Appendix C; available online at www.redjournal.org), leading to large uncertainties in the odds ratios for those covariates. The FDA models featured large uncertainties for the odds ratios of clinical covariates that were highly correlated with other covariates or which applied to small numbers of patients. It should be noted that the regression coefficients of the clinical covariates were not penalized in the FDA models.

There have been very few previous attempts to apply FDA to NTCP modeling (15, 48, 49). These have used either spline basis functions (48, 49) or FPCA (15). To our knowledge, this study represents the first application of FPLS to NTCP modeling. Many previous NTCP modeling studies have not addressed the problem of the high uncertainties of the model regression coefficients caused by multicollinearity. Investigators who have recognized this limitation have avoided the multicollinearity problem by reducing the data describing heterogeneous dose distributions to simple summary metrics, such as mean or maximum dose. However, this leads to suboptimal recommendations for RT planning. For example, using mean dose to optimize or assess RT plans gives equal weight to all dose levels, whereas preferentially minimizing the volume of an OAR receiving high doses rather than low doses is, in fact, likely to result in a lower toxicity incidence.

A limitation of our approach is that, as the technique is an empirical data—driven method, there are decreases in the weighting function describing the relative importance of each dose level with increasing dose, which does not have a biophysical rationale. This should be carefully considered when interpreting dose-response associations from these components. This limitation could be overcome through adopting a Bayesian approach whereby prior knowledge is provided to the model dictating that, with increasing dose level, the weighting function can only remain constant or increase and not decrease. Mathematically, this would take the form of a monotonically increasing prior function (48). The slight difference in the dysphagia scoring systems between the training and external validation cohorts may have reduced the performances of the models on external validation. However, the models performed at least as well on external validation as internal validation. The relatively small size of the external validation cohort should also be considered a potential limitation.

In the future, FPCA or FPLS could be applied to the 3D dose distribution (rather than the DVH) (15), either to a single OAR or to the entire dose grid, encompassing multiple OARs. This would allow associations between spatial aspects of the dose distribution and toxicity to be explored. This would require accurate mapping of the 3D dose distributions onto a common reference.

## Conclusions

FPC-LR and FPLS-LR models of severe acute mucositis had marginally better discrimination than PLR on internal validation. FDA models of dysphagia had marginally improved discrimination and substantially superior calibration compared with PLR on external validation, indicating potential advantages for clinical decision support. FPCA and FPLS enable robust estimates of dose-response associations in the context of correlated dose data. This permits understanding of the most beneficial OAR dose-volume levels, Vx to reduce in RT planning. Minimizing the volumes of the oral cavity and pharyngeal mucosa receiving high and intermediate doses is expected to reduce the incidence of severe acute mucositis and dysphagia. We recommend that FDA methods be applied to future NTCP modeling studies.

## Supplementary Material

1

## Figures and Tables

**Table 1:** Predictive performance of mucositis and dysphagia models on internal validation (corrected for optimism by bootstrapping with 2000 replicates) and external validation (for dysphagia models)

Model	AUC	Brier score	Calibration slope	Calibration intercept
Mucositis				
PLR	0.65	0.21	1.6	−0.67
FPC-LR	0.69	0.19	0.45	0.47
FPLS-LR	0.67	0.20	0.40	0.49
Dysphagia*				
PLR	0.74/0.81	0.20/0.18	1.2/2.5	−0.15/−0.96
FPC-LR	0.76/0.83	0.19/0.18	0.59/0.79	0.21/−0.04
FPLS-LR	0.75/0.83	0.20/0.18	0.56/0.79	0.22/0.00

*Abbreviations:* AUC = area under receiver operating characteristic curve; FPC-LR = functional principal componentelogistic regression; FPLS-LR = functional partial least squares—logistic regression; PLR = penalized logistic regression.

* For the dysphagia models, the metrics of predictive performance are given as internal validation/external validation.

**Table 2:** Odds ratios for penalized logistic regression models

Covariate	Mucositis model		Dysphagia model	
	Odds ratio	95% CI	Odds ratio	95% CI
Intercept	2.512	0.016-12.43	0.360	0.007-2.583
Male	1.000	1.000-2.554	1.000	1.000-1.945
Age	1.000	0.971-1.006	1.000	0.980-1.000
Definitive RT	1.000	0.110-1.000	1.000	0.544-1.000
Induction chemotherapy	1.000	0.410-1.166	1.000	1.000-2.089
Cisplatin	1.000	1.000-3.464	1.277	1.000-3.230
Carboplatin	1.000	0.361-4.015	1.000	1.000-4.278
Cis-carbo	1.000	0.136-1.769	1.000	0.989-2.930
Hypopharynx or larynx	1.000	1.000-14.71	1.000	1.000-2.203
Nasopharynx or nasal cavity	1.000	0.905-6.190	1.000	0.247-1.000
Unknown primary	1.000	0.022-1.000	1.000	0.945-1.210
Parotid	0.814	0.231-2.546	0.600	0.208-1.000
V020	1.000	1.000-1.119	1.000	1.000-1.031
V040	1.000	0.891-1.000	1.000	1.000-1.014
V060	1.000	1.000-1.032	1.000	1.000-1.003
V080	1.000	1.000-1.050	1.000	1.000-1.023
V100	1.000	0.934-1.000	1.000	1.000-1.029
V120	1.000	1.000-1.084	1.019	1.000-1.044
V140	1.000	0.917-1.000	1.000	1.000-1.019
V160	1.000	1.000-1.038	1.000	1.000-1.011
V180	1.002	1.000-1.085	1.000	0.997-1.009
V200	1.000	0.949-1.007	1.000	1.000-1.019
V220	1.000	1.000-1.098	1.008	1.000-1.031
V240	1.000	0.616-1.154	1.000	1.000-1.025
V260	1.000	1.000-1.000	1.000	1.000-1.000

*Abbreviations*: Cis-carbo = 1 cycle of cisplatin followed by 1 cycle of carboplatin; RT = radiation therapy; 95% CI = 95% confidence interval calculated by bootstrapping model fitting with 2000 replicates; V*x* = volume of organ receiving *x* cGy of radiation per fraction.

**Table 3:** Odds ratios for functional principal component—logistic regression models

Covariate	Mucositis model		Dysphagia model	
	Odds ratio	95% CI	Odds ratio	95% CI
Intercept	12.89	1.035-1.734×10^9^*	1.616	0.142-77.46
Male	1.535	0.637-4.088	1.675	0.533-4.880
Age	0.991	0.951-1.029	0.988	0.943-1.027
Definitive RT	0.254	2.679×10^−9^-1.773	0.997	0.080-7.541
Induction chemotherapy	0.487	0.070-1.960	1.100	0.210-7.670
Cisplatin	2.251	0.745-9.540	4.255	1.077-19.86*
Carboplatin	1.320	0.142-7.314×10^7^	4.429	0.685-8.332×10^7^
Cis-carbo	0.311	7.815×10^−9^-2.531×10^7^	2.238	0.319-4.587×10^7^
Hypopharynx or larynx	4.371	0.512-143.9	1.723	0.193-1.881×10^7^
Nasopharynx or nasal cavity	2.370	0.308-1.096×10^8^	0.263	0.026-1.223
Unknown primary	0.136	3.042×10^−9^-3.707	0.859	0.077-3.876×10^6^
Parotid	1.387	0.103-40.37	1.135	0.068-18.72
DVH FPC1	0.997	0.993-1.007	0.996	0.990-1.008
DVH FPC2	1.003	0.992-1.009	-	0.992-1.003
DVH FPC3	-	0.996-1.003	-	0.995-1.000
DVH FPC4	-	0.987-1.010	-	0.991-1.006
DVH FPC5	-	0.971-1.033	-	0.991-1.006

*Abbreviations*: Cis-carbo = 1 cycle of cisplatin followed by 1 cycle of carboplatin; DVH FPC*x* = functional principal component *x* of dose-volume histogram data; RT = radiation therapy; 95% CI = 95% confidence interval calculated by bootstrapping model fitting with 2000 replicates.

The sign of the functional principal component loadings is arbitrary, so the fact that the odds ratios are <1 does not indicate that there is an inverse correlation between RT dose and severe toxicity.

* Statistically significant at α = .05 level.

**Table 4:** Odds ratios for functional partial least squares—logistic regression models

Covariate	Mucositis model		Dysphagia model	
	Odds ratio	95% CI	Odds ratio	95% CI
Intercept	12.90	0.961-2.424×10^10^	1.634	0.128-104.4
Male	1.539	0.620-4.757	1.661	0.472-4.719
Age	0.991	0.947-1.033	0.988	0.942-1.029
Definitive RT	0.260	7.707×10^−11^-1.245	0.975	0.046-7.831
Induction chemotherapy	0.484	0.064-2.442	1.100	0.222-7.866
Cisplatin	2.246	0.728-11.33	4.235	1.083-20.88*
Carboplatin	1.315	0.110-1.051×10^8^	4.393	0.580-8.424×10^7^
Cis-carbo	0.313	8.668×10^−9^-3.303×10^7^	2.245	0.324-4.247×10^7^
Hypopharynx or larynx	4.169	0.506-484.8	1.677	0.168-1.998×10^7^
Nasopharynx or nasal cavity	2.336	0.350-1.457×10^8^	0.266	0.028-1.250
Unknown primary	0.132	2.020×10^−9^-95.47	0.903	0.092-2.895×10^6^
Parotid	1.408	0.097-56.81	1.196	0.071-27.80
DVH FPLS1	1.004	1.002-1.017*	1.005	1.001-1.016*
DVH FPLS2	1.002	1.000-1.047	-	1.000-1.041
DVH FPLS3	-	1.000-1.110	-	1.000-1.009
DVH FPLS4	-	1.000-1.107	-	1.000-1.009
DVH FPLS5	-	1.000-1.085	-	1.000-1.009

*Abbreviations:* Cis-carbo = 1 cycle of cisplatin followed by 1 cycle of carboplatin; DVH FPLS*x* = functional partial least squares component *x* of dose-volume histogram data; RT = radiation therapy; 95% CI = 95% confidence interval calculated by bootstrapping model fitting with 2000 replicates.

* Statistically significant at α = .05 level.

## Summary

Normal tissue complication probability modeling using logistic regression (LR) suffers from bias and uncertainty in the presence of highly correlated radiation therapy dose data. Consequently, robust estimates of the dose levels most strongly associated with toxicity and, potentially, predictive performance are limited. To overcome this limitation, functional data analysis, which describes the dose-volume histogram as a continuous curve, was applied to modeling of severe acute mucositis and dysphagia and compared with LR. Functional data analysis models showed slightly better predictive performance and more robust dose-response estimates than LR.
