# Supplementary material for: Functional Data Analysis Applied to Modeling of Severe Acute Mucositis and Dysphagia Resulting From Head and Neck Radiation Therapy
Source: Int J Radiat Oncol Biol Phys. Author manuscript; Available in PMC 2017 Oct 23. (PMC5653218; doi:10.1016/j.ijrobp.2016.08.013)
Supplement: 1 [file NIHMS895369-supplement-1.pdf]

Appendix A – Summary of the data sets used

Appendix table 1: Data sets included in the modeling.

Trial	Patients available	Primary disease site	Radiation therapy technique	Radiation therapy dose-fractionation	Concurrent chemotherapy
**COSTAR (Phase III, multicentre; ISRCTN 81772291)**	78	Parotid gland	Unilateral; conventional, IMRT	65 Gy / 30 # (definitive RT), 60 Gy / 30 # (post-operative RT)	No
**PARSPORT (Phase III, multicentre) [20]**	71	Oropharynx, hypopharynx	Bilateral; conventional, IMRT	65 Gy / 30 # (definitive RT), 60 Gy / 30 # (post-operative RT)	No
**Dose Escalation (Phase II, single centre) [21]**	30	Larynx, hypopharynx	Bilateral; IMRT	67.2 Gy / 28 #, 63 Gy / 28 #	Yes
**Midline (Phase II, single centre) [22]**	117	Oropharynx	Bilateral; IMRT	65 Gy / 30 # (definitive RT), 60 Gy / 30 # (post-operative RT)	Yes
**Nasopharynx (Phase II, single centre) [23]**	36	Nasopharynx	Bilateral; IMRT	65 Gy / 30 # (definitive RT), 60 Gy / 30 # (post-operative RT)	Yes
**Unknown Primary (Phase II, single centre) [24]**	19	Unknown primary	Bilateral; IMRT	65 Gy / 30 # (definitive RT), 60 Gy / 30 # (post-operative RT)	Yes
**Washington University**	90	Oral cavity, nasal cavity, nasopharynx, oropharynx, hypopharynx, larynx, parotid gland, unknown primary	Bilateral, unilateral; IMRT	70 Gy / 35 #, 66 Gy / 33 #, 60 Gy / 30 #	Yes, no

IMRT - intensity-modulated radiotherapy; # - fractions; RT – radiation therapy; Unilateral – treatment delivered to ipsilateral parotid bed only; Bilateral – treatment delivered to ipsilateral and contralateral mucosa of relevant subsite (e.g. nasopharynx, oropharynx or larynx).

Appendix B – Strategy for handling missing data

If weekly toxicity data are incomplete this can lead to assignment of an incorrect peak toxicity grade. For example, a patient has grade 1 toxicity for weeks 1 to 3, grade 2 toxicity for weeks 4 and 5, missing toxicity week 6 and 1 week following treatment and grade 2 toxicity from 2 weeks following RT to 8 weeks following RT. They would be assigned a peak grade of 2. However, they may, in fact, have experienced grade 3 toxicity, which was not scored, as they were unable to attend their follow-up appointments. This would introduce an error into the analysis. As this type of error can only lead to peak toxicity being under-scored and not over-scored it could introduce bias. Therefore, in an attempt to reduce bias at the expense of statistical power, patients with any missing toxicity scores and a peak score below 3 were excluded from the analysis. Missing toxicity data were not imputed as many patients (with full toxicity data) with peak toxicity of grade 3 were only scored as grade 3 for one week. We previously investigated the effects of imputing missing toxicity measurements, where there were non-consecutive missing values and found that this made little difference [25]. Patients with some missing toxicity measurements, but at least one measurement scored as grade 3 were included as they must have a peak grade of 3 or higher. It should be noted that retaining patients with missing data, but having a peak grade of 3 skews the apparent incidences of peak toxicity grades. There was a general trend that data were more likely to be missing around the middle to the end of treatment, which was when the peak grade of toxicity tended to occur.

It should be noted that our approach to handling missing data might still result in bias. Where there are missing data there is always a risk of bias whichever method for handling missing data is used. This is particularly true where the data are not missing at random as is suggested by the pattern of missing data in this data set. Ultimately, the performance of the model, including any bias introduced by the missing data handling strategy, is assessed by external validation. The external validation dataset had no missing PEG-dependence data.

Appendix C – Comparison of clinical covariate data between training and external validation data sets

Appendix table 2: Clinical covariate data in the training and external validation data sets.

Covariate	$n_{training}$ (%)	$n_{validation}$ (%)
Definitive RT	148 (86)	44 (49)
Male	114 (66)	68 (76)
Induction chemotherapy	94 (54)	21 (23)
No concurrent chemotherapy	82 (47)	46 (51)
Cisplatin	66 (38)	28 (31)
Carboplatin	14 (8)	0 (0)
Cisplatin/Carboplatin	11 (6)	0 (0)
Hypopharynx/Larynx	24 (14)	25 (28)
Oropharynx/Oral cavity	87 (50)	41 (46)
Nasopharynx/Nasal cavity	18 (10)	15 (17)
Unknown primary	10 (6)	3 (3)
Parotid gland	34 (20)	6 (7)
Covariate	$median_{training}$ (range)	$median_{validation}$ (range)
Age	59 (23 - 88)	58 (21 – 87)

Appendix D – Pharyngeal mucosa organ at risk

Appendix figure 1 displays an example of the pharyngeal mucosa contouring technique employed.

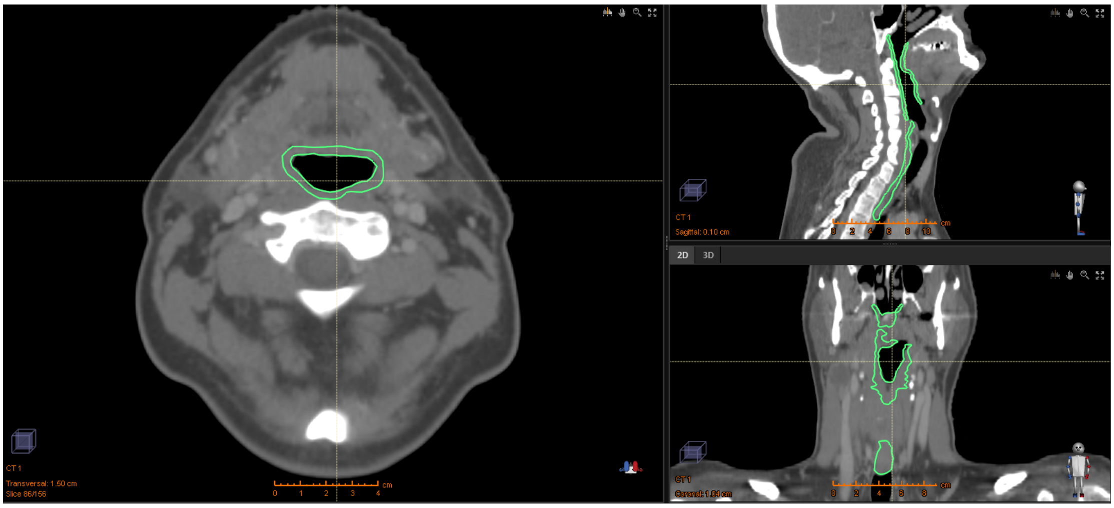

Appendix figure 1: Axial (left), sagittal (top right) and coronal (bottom right) views of an example of the pharyngeal mucosa structure used.

The cranial extent of the structure is the roof of the nasopharynx and the caudal extent is the level of the suprasternal notch. The pharyngeal constrictor muscles were included in the structure, as the mucosa cannot be easily differentiated from the muscles on a planning CT scan. Further detail is given in [46]. Note that in the present study the pharyngeal mucosa structure was extended inferiorly compared with the structure described in [46] as for some patients the superior esophagus received a substantial dose, which may have contributed to dysphagia. Most patients in the training data cohort were treated with extended neck positioning, in order to reduce oral cavity doses. Patients in the external validation cohort were treated with a neutral neck position. Contouring the structure took approximately 5 minutes per patient.

Appendix E – Correlation matrices

Appendix figures 2 and 3 show correlation matrices for the data used in the mucositis and dysphagia modeling, respectively.

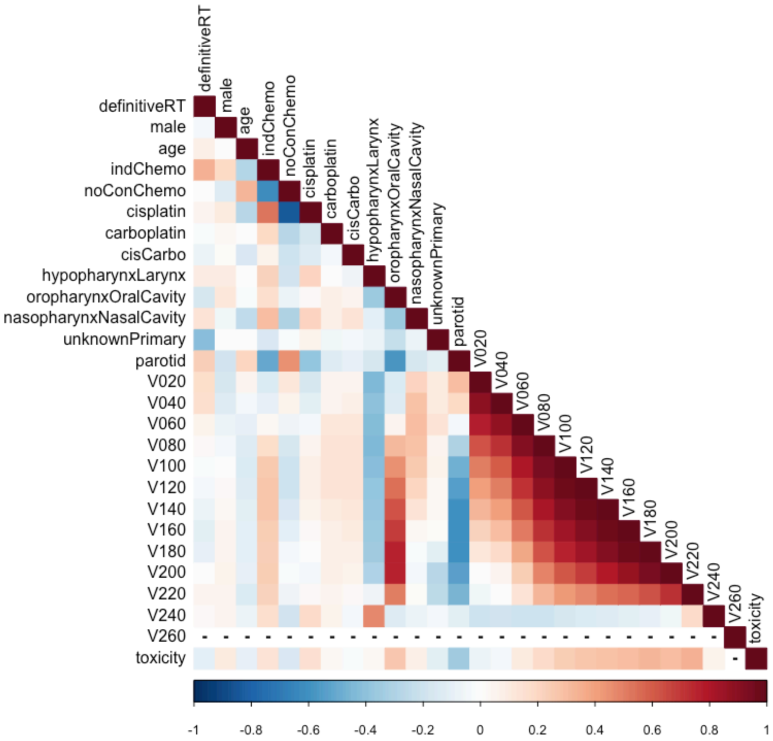

Appendix figure 2: Spearman correlation matrix for data used in the mucositis PLR model. The dashes for V260 represent a standard deviation of 0 as no patients received a fractional dose of 260 cGy or higher to the extended oral cavity. definitiveRT – definitive radiation therapy; indChemo – induction chemotherapy; noConChemo – no concurrent chemotherapy; cisCarbo – one cycle of cisplatin followed by one cycle of carboplatin; Vx – volume of extended oral cavity receiving a fractional dose of x cGy or higher; toxicity – severity of mucositis (non-severe = 0, severe = 1).

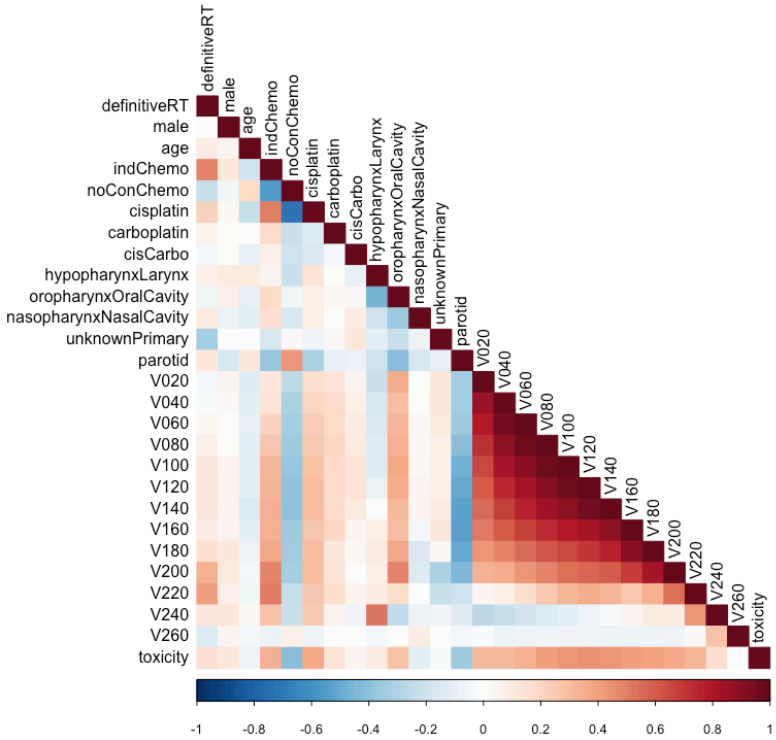

Appendix figure 3: Spearman correlation matrix for data used in the dysphagia PLR model. definitiveRT – definitive radiation therapy; indChemo – induction chemotherapy; noConChemo – no concurrent chemotherapy; cisCarbo – one cycle of cisplatin followed by one cycle of carboplatin; Vx – volume of pharyngeal mucosa receiving a fractional dose of x cGy or higher; toxicity – severity of dysphagia (non-severe = 0, severe = 1).
